# Supplementary material for: Length of hospital stay and its associated factors among women who gave birth by cesarean section in general hospitals of Sidama region, Ethiopia
Source: PLOS Glob Public Health. 2025 Jun 5;5(6):e0004582. doi: 10.1371/journal.pgph.0004582 (PMC12140221; doi:10.1371/journal.pgph.0004582)
Supplement: Attachment — Submitted filename: Response to Reviewers.docx [file pgph.0004582.s004.docx]

**Date: 11 May 2025**

# **Authors Response to Editor and Reviewers**

#### Manuscript Id: PGPH-D-25-00744

**Manuscript Title:** Length of Hospital Stay and Its Associated Factors among Women Who Gave Birth by Cesarean Section in General Hospitals of Sidama Region, Ethiopia

| S.No | Editor Comments | Authors Response | Line No &Page No |
| --- | --- | --- | --- |
| #1 | Please ensure that the Title in your manuscript file and the Title provided in your online submission form are the same. | Dear Editor, we have verified that the title in the manuscript file matches the title provided in the online submission form. |  |
| #2 | Your manuscript is missing the following sections: Introduction. Please ensure these are present, and in the correct order, and that any references to subheadings in your main text are correct. An outline of the required sections can be consulted in our submission guidelines here | Dear Editor, in our previously submitted manuscript, we had included the 'Introduction' section under the 'Background' subsection. We have now corrected this based on your comment. | L 41, Page 3 |
| #3 | We have noticed that you have uploaded Supporting Information files, but you have not included a list of legends. Please add a full list of legends for your Supporting Information files after the references list. | Dear Editor, the legends for Supporting Information files have been included as a subsection after the references. | L 329-31, Page 21 |
| #4 | Please review your reference list to ensure that it is complete and correct. If you have cited papers that have been retracted, please include the rationale for doing so in the manuscript text, or remove these references and replace them with relevant current references. Any changes to the reference list should be mentioned in the rebuttal letter that accompanies your revised manuscript. If you need to cite a retracted article, indicate the article’s retracted status in the References list and also include a citation and full reference for the retraction notice. | Dear Editor, we confirm that no retracted references were included in our reference list. All references were cited in their appropriate places. |  |
| Reviewer 1 Comments | | | |
| S.No | **Reviewer 1 Comments** | **Authors Response** | **Line No &Page No** |
| #1 | Method: Some details of the hospitals where CS are conducted is needed in the methods section on study area and period. Details such as number of deliveries and C-section rates is required to get an overview of the case load in these ??six hospitals. It needs to be clear that the data is collected from tertiary hospitals where C section facility is available. | Dear Reviewer, during the data collection period, there were six general hospitals in the region. Since then, one primary hospital has been upgraded to a general hospital, bringing the current total to seven government general hospitals. As noted in the study area description, all government general hospitals provide delivery services, and 4,082 women delivered via cesarean section in these facilities in 2024. | L 98-100, Page 5 |
| #2 | If you can add a prevalence rate of C-sections in the Sidama region, as it would be a useful addition to the results section. | Dear Reviewer, we did not collect data on the total number of deliveries performed in general hospitals, as our primary objective was to assess hospital stay following cesarean sections. Therefore, we are unable to report the prevalence rate of C-sections in the Sidama region. |  |
| #3 | If possible, information on the cost of C-sections, including the hospital stay, is required since it can also influence the decision to discharge early. | Dear Reviewer, as clearly indicated in the revised manuscript's study area description sub-section, all public hospitals in the region provide cesarean section services free of charge. | L 98-100, Page 5 |
| Reviewer 2 Comments | | | |
| S.No | **Reviewer 2 Comments** | **Authors Response** | **Line No &Page No** |
| #1 | It is unclear why the sample size was calculated based on folate intake adequacy, as this variable was not assessed in the study (page 5). | Dear Reviewer, the authors estimated the sample size for this study based on data from a previous study conducted in Sudan on the length of hospital stay after cesarean section. The phrase ‘folate intake adequacy’ was inadvertently inserted into the manuscript due to a typographical error. | L 108, Page 5 |
| #2 | Explain how systematic sampling was implemented (page 6). | Dear Reviewer, we understand your concern regarding the application of systematic sampling method and inclusion of every mother in our study. This approach was guided by the systematic sampling method, which led us to include all post-CS mothers during the data collection period.  Given our available resources, we planned to complete data collection within two months. To determine the sampling interval constant, we reviewed postnatal registration logbooks in the selected hospitals to estimate the number of mothers who delivered via cesarean section (CS) during that period. Based on the records, we found that 558 mothers had given birth by CS in the previous two months.  Using the formula for the sampling interval constant (**K = Total CS mothers / Sample size**), we calculated **K = 558/503 = 1.12**. Since we rounded **K** down to **1**, we included every mother who delivered by CS in the selected hospitals during the study period. | L 116-124, Page 6 |
| #3 | Which obstetric complications were considered in the study? (page 6) | Dear Reviewer, we used the term 'current obstetric complications' to refer to pregnancy-related conditions that women developed either before admission to the selected hospitals or those diagnosed prior to cesarean section. The complications included in this category were: hypertensive disorders of pregnancy, antepartum haemorrhage, gestational diabetes mellitus, premature rupture of membranes, congenital anomalies, neonatal loss, and intrauterine growth restriction.  We grouped these conditions into a single variable ('current obstetric complications') because the number of cases for each individual complication was insufficient for separate analysis. | L 208-210, Page 12 |
| #4 | Could you please clarify what is meant by 'grand multipara? (Page 10, Table 2). | Dear reviewer, in this study, we considered ‘parity’ as an independent variable and classified mothers into three categories: Primipara (a woman who has given **birth once to a viable fetus**), Multipara (a woman who has given birth **two or more times to viable fetuses**), and Grand Multipara (**a woman who has given birth five or more times to viable fetuses**). | L 76-8, Page 9, Table 2 |
| #5 | Which chronic medical conditions were included in the study? (Page 10, Table 2). | Dear Reviewer, chronic medical disease considered in this study were chronic hypertension without proteinuria, cardiovascular diseases, pre-gestational diabetes mellitus, asthma, chronic pulmonary diseases. | L 185-7, Page 10 |
| #6 | Kindly specify the units for low birth weight (e.g., less than ___ g and up to 2,500 g) and normal birth weight (Table 3). | Dear Reviewer, the units for low birth weight (less than 2,500 grams) and normal birth weight (≥2,500 grams) are specified in Table 3, as requested. | L 190, Page 10  Table 3 |
| #7 | Could you please clarify what is meant by low and normal APGAR scores? (Table 3). | Dear Reviewer, based on the five APGAR (Appearance, Pulse, Grimace, Activity, Respiration) score assessment criteria at **five minutes after birth**, we classified scores as **low (≤6)** or **normal (7–10)**. This classification was also incorporated into the **revised document**. | L 191, Page 10  Table 3 |
| #8 | Please describe the obstetric complications that were included in the study (Table 3). | Dear Reviewer, in this study we considered both the 'current obstetric problems' noted in comment #3 above and some complications occurred during/post cesarean delivery procedure, including anaesthesia complications, surgical site infections, cesarean hysterectomy, low APGAR scores, and neonatal admission to the Intensive Care Unit. All of these were clearly described in the sub-sections 'Neonatal Factors' (Table 3) and 'Obstetric Factors' (Table 4). | L 208-210, Page 12  Table 2 |
| #9 | Can author explain how has recall bias potentially influenced the outcomes reported in this study? (page 17) | Dear Reviewer, we acknowledge that recall bias could influence the outcomes of this study, as the data were collected from postpartum women. For example, opioids or sedatives administered after cesarean delivery may impair short-term memory, potentially leading to underreporting of events or inaccurate timing of recall. Additionally, stress resulting from emergency cesarean sections or birth trauma could cause overreporting of negative experiences or memory gaps (e.g., forgetting specific events) |  |
| #10 | I think, this should be the median length of hospital stay. (page 17, conclusion). | Dear Reviewer, yes - as you noted, it is the median length of hospital stay. We have made the correction accordingly in the revised document. | L 289, Page 17 |
